# Contamination of livestock due to the operation of a small waste incinerator: a case incident in Skutulsfjörður, Iceland, in 2010

**DOI:** 10.1186/1751-0147-54-S1-S4

**Published:** 2012-02-24

**Authors:** Thorhallur I Halldorsson, Guðjón Atli Auðunsson, Rannveig Guicharnaud, Ólafur R Dýrmundsson, Sigurður Örn Hansson, Kjartan Hreinsson

**Affiliations:** 1 Unit for Nutrition Research, Landspitali University Hospital, Iceland; 2 Faculty of Food Science and Nutrition University of Iceland, Reykjavík, Iceland; 3 Innovation Center Iceland, 112 Reykjavík, Iceland; 4 Agricultural University of Iceland, Hvanneyri, Iceland; 5 The Farmers Association of Iceland, Reykjavík, Iceland; 6 Icelandic Food and Veterinary Authority (MAST), Iceland

## Abstract

**Summary:**

**Background**

In 2010 contamination by dioxins and dioxin-like PCBs was detected in milk and meat in the valley Engidalur situated at the bottom of a fjord (Skutulsfjörður) in North West Iceland. The valley is narrow and surrounded by high mountains resulting in prevailing calm weather. The contamination was traced to a small municipal waste incinerator operating in the valley. Annual agricultural production in Engidalur was modest (≈6 tons of meat and 45 tons of milk). The Icelandic Food and Veterinary Authority conducted a series of measurements examining the contamination and the results are reported in this paper.

**Results**

Earlier inspection of the waste incinerator had shown dioxin levels in fly ash of 2.1 ng I-TEQ/m^3^, which exceeded the EU maximum limit of 0.1 ng I-TEQ/m^3^. Late in 2010 routine inspection found 4.0 pg WHO-TEQ/g for PCDD/Fs and 7.4 pg total WHO-TEQ/g fat in one milk sample from a farm in Engidalur; levels exceeding the EU maximum limits of 3.0 and 6.0 pg WHO-TEQ/fat for dairy fat, respectively. These results were confirmed in an additional milk sample. Elevated levels exceeding the maximum limits were also observed in one out of two beef samples collected from the farm (4.7 pg WHO-TEQ/g for dioxins and 12.3 pg total WHO-TEQ/g fat). Elevated levels in lamb and ewe meat were also observed but concentration varied greatly, reflecting different migration routes of animals during summer grazing and different sources of hay used during winter. A composite sample of hay from Engidalur had levels of PCDD/Fs of 0.85 pg WHO-TEQ/g and 1.36 pg total WHO-TEQ/g; levels that were marginally, but not significantly, above the EU maximum limit of 0.75 pg WHO-TEQ/g and 1.25 pg WHO-TEQ/g, respectively.

**Conclusions**

Operation of a small municipal waste incinerator, not fulfilling modern standards, may lead to elevated levels of dioxins in local livestock.

## Introduction

Polychlorinated dibenzo-p-dioxins (PCDDs) and dibenzofurans (PCDFs), referred to as dioxins, are formed as unintentional by-products in various industrial processes including waste incineration. Dioxins may also be formed by natural processes like natural fires of vegetated areas but these sources are usually of much less importance than the anthropogenic ones. Other compounds possess dioxin-like properties, notably some polychlorinated biphenyls (PCBs), *i.e.* the dioxin-like PCBs (DL-PCBs). Based on adverse developmental effects observed in laboratory animals, the tolerable weekly intake of dioxins and dioxin-like PCBs for humans has been estimated to be 14 pg WHO1998-TEQ/kg b.w. [1]. Dioxins and dioxin-like PCBs are also classified as human carcinogens [2] and human exposure to these contaminants has been associated with a number of other adverse health effects [3-5]. The toxicity of dioxins and dioxin-like compounds is quantified in terms of toxic equivalents (TEQ) calculated by way of toxic equivalent factors (TEFs), which rank the different congener’s relative toxicity towards the most toxic dioxin, 2,3,7,8-TCDD, which has a TEF equal to one [6]. There are 210 possible congeners of PCDD/Fs and 209 congeners of PCBs of which seventeen and twelve have TEFs, respectively. Dioxins and PCBs are very persistent compounds with elimination half-life in humans ranging from 1 to >20 years [7]. Dioxins and PCBs accumulate in fat and biomagnify in the food web of aquatic and terrestrial animals. As a result, foods of animal origin usually account for more than 90% of human exposure [8].

Levels of dioxins and PCBs in humans have decreased to less than 20% of peak levels that were observed in the early 1970s [9]. This decrease is likely to reflect the reduction in use of organochlorine compounds and introduction of strict legislation on emissions from for example incineration processes [10]. Stipulation of maximum and action limits for dioxins and dioxin-like PCBs in food and feed may also have played some role [11]. Despite considerable emphasis of reducing dioxins and dioxin-like PCBs in food and feed, incidents of accidental food contamination are occasionally reported. In some cases contamination has occurred when contaminated oils or additives are accidentally mixed with animal feed [12-14]. Direct contamination of livestock through intake of contaminated plants and soils appears to be less frequent. This is mainly due to the chemical characteristics of dioxins which are characterized by low vapor pressure, low aqueous solubility and strong sorption to organic matter leading to limited water leaching or plant uptake [15,16]. Incident of food contamination through uptake of contaminated plants and soils has, however, recently been reported in the vicinity of Naples, Italy, where the suspected source was illegal open burning of waste [17].

In late 2010, elevated levels of dioxins were detected in milk, beef and lamb in Northwestern Iceland. The contamination was localized to a narrow valley, Engidalur, at the bottom of a fjord, Skutulsfjörður. A small municipal waste incinerator was situated in the valley. Since the contamination was discovered, the Icelandic Food and Veterinary Authority has monitored the area and the results of that work are reported in this paper.

## Materials and methods

### Case setting

**Figure 1:**
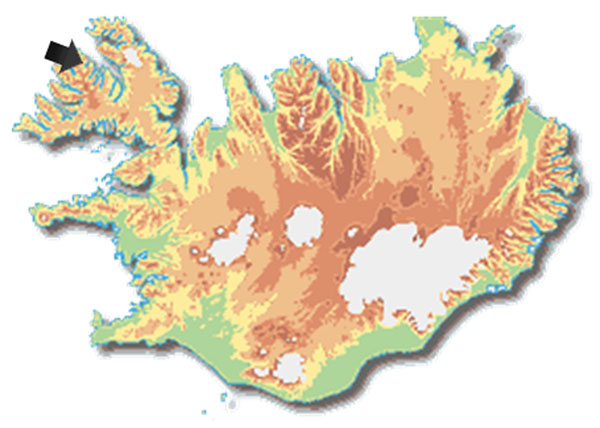
The location of Skutulsfjörður í W- Iceland is indicated by the black arrow.

**Figure 2:**
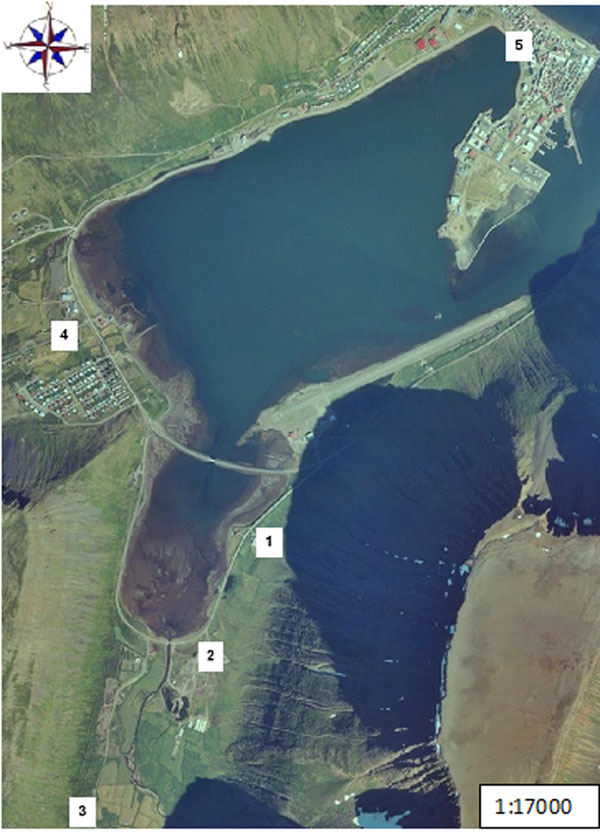
Aerial photograph of Skutulsfjörður. The numbers give the location of the (1) waste incinerator, (2) nearby sheep farm in Engidalur, (3) nearby dairy farm in Engidalur; and (4,5) local town of Ísafjörður.

In December 2010 an internal control revealed non-compliant levels of dioxin and dioxin-like PCBs in milk from Skutulsfjörður. The sample was a composite sample form the only dairy farm in the fjord, located in a narrow valley called Engidalur. The valley is situated in the bottom of the fjord with high mountains on each side. Calm weather is dominant in the valley. The dairy farm produced approximately 45 tons of milk annually. Additional agricultural production in Engidalur was approximately 2 tons of meat from the dairy farm (beef and sheep) and 4 tons of sheep meat produced by a nearby farm in the valley. The location of Skutulsfjörður in Iceland is shown in Figure 1. The source of the contamination was a small waste incinerator processing roughly 3000 tons waste per year from the local community. The incinerator was operating within a 2km radius of the two farms. An aerial photograph showing the location of the incinerator and the two farms is given in Figure 2. A previous control inspection of the waste incinerator in 2007 had shown dioxins levels in fly ash of 2.1 ng I-TEQ/m^3^, which is proximately 20 times higher than the maximum limit of 0.1 ng I-TEQ/m^3^ set by the current EU incinerator directive [18]. This control from 2007 was the only inspection carried out since the operation of the incinerator started in 1995. The incinerator had been operating on a dispensation from the EU incinerator directive. Prior to the current incident, the results from the 2007 inspection were neither made publicly available nor had they been reported to the Icelandic Food and Veterinary Authority.

### Collection of samples

As the dairy herd was localized at the farm and fed on hay from the valley, one additional composite milk sample and two samples of beef were considered sufficient for examining contamination at the farm. A composite sample of hay harvested from the farm during the summer was also collected. For control, and to examine the spread of the contamination, 2 composite milk samples were collected from nearby fjords. Average values from a previous survey conducted in 2003-2004 were also used for comparison (n=10).

Evaluating contaminant levels in sheep were hampered by several factors. Firstly the sheep from the dairy farm were fed on hay from Engidalur during winter but grazed in a nearby fjord on the other side of the mountains during the summer. Sheep form the other farm in Engidalur received hay during the winter that was harvested outside Engidalur but the herd grazed in the valley during summer. For examining the contaminant levels in sheep a total of 11 meat samples from Engidalur were collected. For control, average values from samples of lambs from the 2003-2004 survey were used (n=5), and an average value for the three control samples of sheep collected in 2011 (n=3). All meat samples collected in Engidalur were from animals that had been slaughtered 2-4 months prior to the incident.

Following up on the current incident, the Environment Agency of Iceland examined the levels of dioxins in soils in Engidalur and surrounding area [19]. Soil samples were collected after the overlying grass turf was removed after which a profile down to 5cm depth was collected [20].

### Chemical analysis

The samples of milk, meat and hay were analysed at the National Food Institute, Technical University of Denmark. The analytical method has been described elsewhere [21]. In short, the requirements of the commission regulation (EC) No 1883/2006 for official control of dioxins and dioxin-like PCBs were followed [22]. Fat extraction was performed by accelerated solvent extraction on ASE300 (Dionex). Quantifcation of dioxins and PCBs was made by gas chromatography and detection by high resolution mass spectrometer (GCHRMS, Trace GC ultra and Finnigan MAT95). The GC was equipped with split/splitless injector and DB5MSDG column (10m pre-column, 60 m, 0.25 mm I.D, film thickness 0.25 μm). Standards were obtained from Cambridge Isotope Laboratories, INC. (USA) and Dr. Ehrenstorfer (Germany). The congeners analyzed were the seven and ten chloro substituted PCDDs and PCDFs, respectively; the four non-ortho PCBs (no. 77, 81, 126 and 169); eight mono-ortho PCBs (no 105, 114, 118, 123, 156, 157, 167 and 189); and the six marker PCBs (no 28, 52, 101, 138, 153 and 180).

The Environment Agency of Iceland was responsible for the soil samples. Samples were dried at 105 °C according to DIN 38414-S2 and sieved for collection of the <2 mm fraction for analysis, which was carried out by Eurofins GfA, Germany.

## Results

Overview of the types of samples collected and description of the sample location is given in Table 1. To examine the spread of the contamination, PCDD/Fs, dioxin-like PCBs and 7-marker PCBs in milk from Engidalur were compared with contaminant levels in samples collected in two nearby fjords. For all sample types (milk, beef, sheep (lambs/ewes), and hay), average values from samples collected in 2003-2004 at various locations around Iceland were also used for comparison. The data of 2003-2004 represent background contamination in Iceland.

**Table 1:** Description and specification of samples

Sample	Year	Type	Location	Description
	
Engidalur	2010-2011	Milk (composite sample), beef, ewes, lamb and hay, soil	Skutulsfjörður, NW Iceland. See Figure 2	Contaminated area. A dairy farm and a small sheep farm located within 2km radius from the waste incinerator
Control A	2010-2011	Milk (composite sample), soil/sediment	Álftafjörður, NW Iceland	Nearby fjord on the other side of the mountains, south east from Engidalur (≈ 10 km direct distance)
Control B	2010-2011	Milk (composite sample)	Önundarfjörður, NW Iceland	Nearby fjord on the other side of the mountains, south west from Engidalur (≈ 10 km direct distance)
Control C	2003-2004	Milk (composite samples), beef, lamb, hay	Different locations in Iceland	Average values from a previous control survey

Both the composite milk samples collected in Engidalur exceeded the EU maximum limits of 3.0 and 6.0 pg WHO-TEQ/fat for PCDD/Fs and the sum of dioxins and dioxin-like PCBs, respectively [11] (Table 2). The two control samples (A and B), taken in nearby fjords, were far below the maximum limits; with levels of PCDD/Fs of around 0.2 pg WHO-TEQ g/fat and the sum of dioxins and dioxin-like PCBs of 0.7 pg WHO-TEQ g/fat. The contaminant levels in the control samples A and B were close to levels previously observed as background levels in Iceland found in the 2003-2004 survey (n=10). The sum of the 7 marker-PCBs in the milk samples were around 5-fold higher in Engidalur compared to the control samples. As expected, elevated levels of PCDD/Fs and dioxin-like PCBs were also observed in hay from Engidalur. Taking analytical precision into account, the observed levels were marginally but not significantly above the maximum levels of 0.75 pg WHO-TEQ/g for PCDD/Fs and 1.25 pg for total WHO-TEQ/g [23]. In comparison, non-detectable levels for most PCDD/Fs-congeners had previously been observed in hay in the 2003-2004 survey.

**Table 2:** Upperbound levels of PCDD/Fs, dioxin-like PCBs and marker PCBs in milk, beef and hay from Engidalur in comparison to control samples

Sample no. and type		PCCD/Fs	DL-PCBs	Total TEQ	Sum 7 PCBs^1^	Comment^2^
		pg WHO-TEQ g/fat			ng g/fat	
Composite milk samples						
M1	Engidalur	3.98	3.44	7,42	9.39	Non-compliant
M2	Engidalur	4.91	5.33	10.24	10.3	Non-compliant
M3	Control A	0.17	0.16	0.33	2.0	Compliant
M4	Control B	0.22	0.53	0.75	2.4	Compliant
M5	Control C^3^	0.14	0.40	0.54	4.40	Compliant
Beef						
B1	Engidalur	4.68	7.63	12.31	21.7	Non-compliant
B2	Engidalur	2.66	2.90	5.56	9.3	Above action level
B3	Control C^4^	0.18	0.39	0.57	1.85	
Composite hay sample		pg WHO-TEQ/g			ng/g	
H1	Engidalur	0.85	0.51	1.36	1.3	Above action level
H2	Control C^5^	0,11	0,12	0.23	3.9	

^1^ PCB number 28, 52, 101, 118, 138, 153 and 180.

^2^ According the COMMISSION REGULATION (EC) No 1881/2006 setting maximum levels for certain contaminants in foodstuffs and COMMISSION RECOMMENDATION (EC) No 88/2006 on the reduction of the presence of dioxins, furans and PCBs in feedingstuffs and foodstuffs.

^3^ Average of 10 samples.

^4^ Average of 3 samples.

^5^ Average of 4 samples for the PCDD/Fs and 2 samples for the sum 7 PCBs (the 7 marker PCBs were not measured in two samples). Levels in all hay samples are based on 12% moisture content.

**Figure 3:**
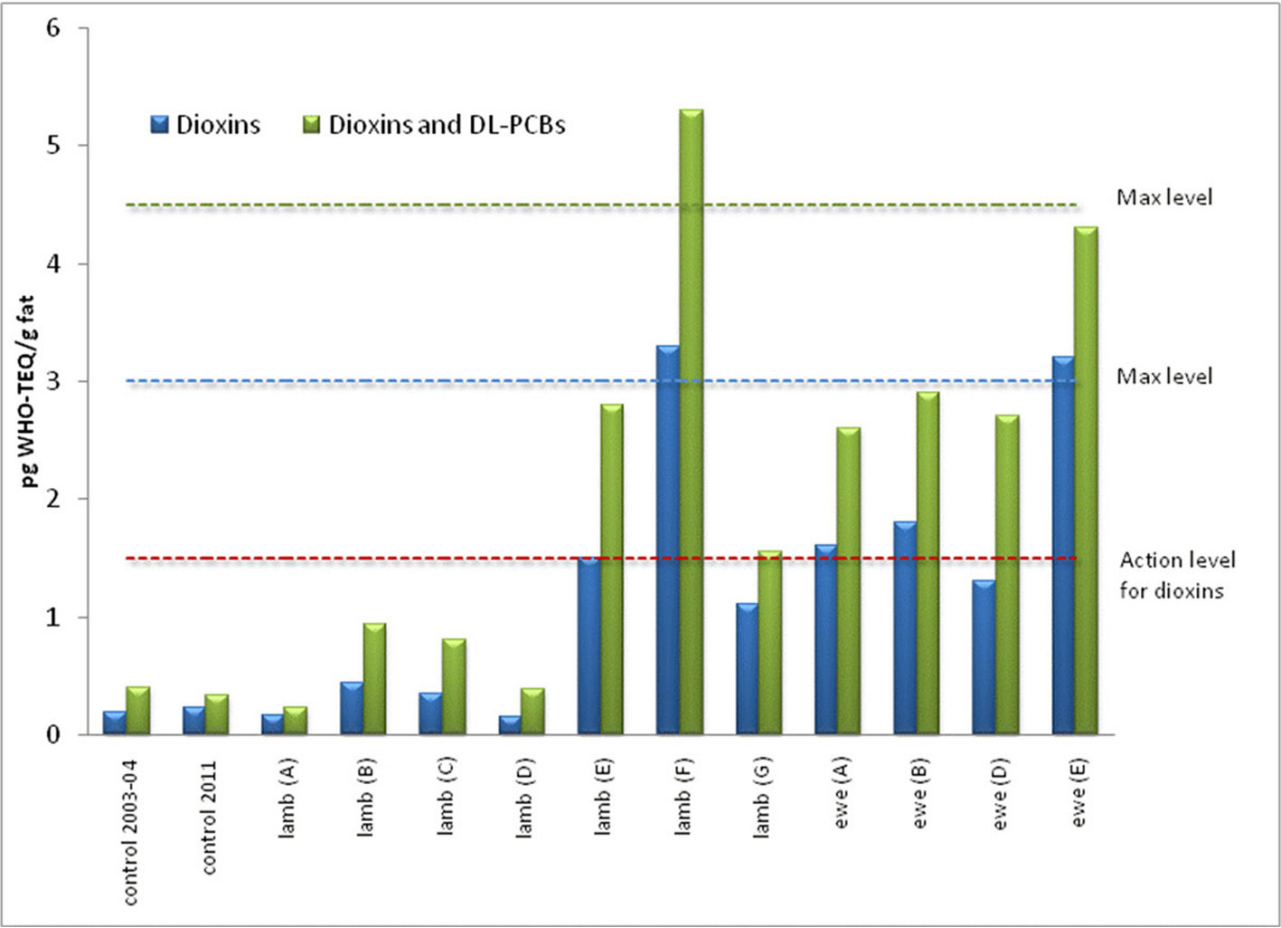
Concentration of PCDD/Fs and total dioxins and dioxin-like PCBs in lamb and ewes from Engidalur 2011. Average values in Icelandic lamb from a previous survey in 2003-2004 (n=5) and control samples taken in 2011 (n=3) are also presented as a comparison. Lamb A-D are lambs from Engidalur that are known to have grazed outside Engidalur prior to slaughtering. Lambs E-G are known to have grazed in Engidalur. The four ewe samples were from Engidalur with ewes A-B and E grazing in the valley during summer but receiving hay from outside the valley during winter. Ewe D received hay from the valley during winter but grazed outside the valley during summer.

The levels of PCDD/Fs and total WHO-TEQ in 11 samples of lambs and ewes from Engidalur are shown in Figure 3. For comparison, average values for samples of lambs from the 2003-2004 survey (n=5) and samples collected in 2011 (n=3) are also shown. Lambs A-D were similar to the control samples. These lambs were born in Engidalur but grazed during their lifespan in a nearby fjord prior to slaughtering. Lambs E-G are known to have grazed, at least partly, in the contaminated valley. Ewes providing samples A-B and E grazed most likely in the valley during summer but these animals received hay from outside the valley during winter. Ewe D is believed to have received hay from the valley during winter but grazed outside the valley during summer.

The congener profiles of PCDD/Fs in the milk and hay samples from Engidalur are shown in Table 3. Both absolute (pg/g) and relative concentrations (% of PCDD/Fs WHO-TEQ) are reported. In short, similar profiles of PCDD/Fs are observed in milk and hay from Engidalur and this profile appears to be different from the relative PCDD/Fs levels in hay from the 2003-2004 survey. A detailed comparison is, however, hampered by many non-detects in the control samples. A more detailed comparison of milk and hay samples is, however, possible for the 7 marker PCBs (Table 4) and the dioxin-like PCBs (Table 5). That comparison reveals a different pattern of marker PCBs in hay from Engidalur compared with hay from the 2003-2004 survey.

**Table 3:** Comparison between different PCDD- and PCDF-congeners in milk and hay form Engidalur with background levels. Both absolute concentrations and the relative contributions of TEQ of each congener are presented.

	Hay (pg /g)^1^		Milk (pg /g fat)		Hay (% of PCDD/Fs WHO-TEQ)		Milk (% of PCDD/Fs WHO-TEQ)	
	Engidalur	Control C^2^	Engidalur	Control C^3^	Engidalur	Control C^2^	Engidalur	Control C^3^
	
2,3,7,8 TCDF	<0.05	0.13	0.29	<0.07	<0.6	12.9	0.7	<6.0
1,2,3,7,8 PeCDDF	0.41	<0.03	0.17	<0.03	2.5	<1.5	0.2	<2.6
2,3,4,7,8 PeCDF	0.15	<0.03	3.18	0.017	29.9	<15.0	36.1	1.5
1,2,3,4,7,8 HxDCF	0.56	<0.03	1.72	<0.02	6.7	<3.0	3.9	<1.7
1,2,3,6,7,8 HxCDF	0.75	<0.03	2.14	<0.02	9.0	<3.0	4.9	<1.7
2,3,4,6,7,8 HxCDF	0.89	<0.03	2.78	<0.05	10.6	<3.0	6.3	<4.0
1,2,3,7,8,9 HxCDF	0.01	<0.03	0.08	<0.08	0.1	<3.0	0.2	<7.0
1,2,3,4,6,7,8 HpCDF	2.60	0,14	0.74	<0.02	3.1	1,4	0.2	<1.7
1,2,3,4,7,8,9 HpCDF	0.24	<0.04	0.15	<0.07	0.3	<0.4	0.0	<6
OCDF	0.99	0.22	0.40	<0.14	0.0	0.0	0.0	<12
2,3,7,8 TCDD	<0.05	<0.02	0.42	<0.03	<5.7	<20.0	9.7	<2.6
1,2,3,7,8 PeCDD	0.19	<0.03	1.48	<0.04	22.7	<30.0	33.7	<3.4
1,2,3,4,7,8 HxCDD	0.15	<0.03	0.61	<0.06	1.8	<3.0	1.4	<5.1
1,2,3,6,7,8 HxCDD	0.28	0.03	1.20	<0.03	3.3	<3.4	2.7	<2.6
1,2,3,7,8,9 HxCDD	0.19	<0.03	0.47	<0.04	0.2	0.3	0.1	<3.4
1,2,3,4,6,7,8 HpCDD	1.70	0.35	1.07	<0.05	0.2	<0.3	0.0	<4.3
OCDD	2.0	1,35	5.96	<0.4	0.0	0.1	0.0	<34

^1^ The results are based on hay with 12% moisture content.

^2^ Average of 4 samples.

^3^ Average of 10 samples.

**Table 4:** Comparison between the different 7 marker PCBs^1^ in milk and hay^2^ form Engidalur with background levels. Both absolute concentration and relative concentrations for each congener are reported.

	Hay (ng/g)		Milk (ng/g fat)		Hay (% of sum 7 PCBs)		Milk (% of sum 7 PCBs)	
	Engidalur	Control C^3^	Engidalur	Control C^4^	Engidalur	Control C	Engidalur	Control C
	
PCB-28	0.11	1.20	0.16	<0.09	8.1	30.5	1.7	<2
PCB-52	0.18	0.76	0.10	<0.04	13.2	19.3	1.0	<1
PCB-101	0.33	0.93	0.22	<0.2	24.3	23.7	2.2	<5
PCB-118	0.31	0.30	4.36	0.7	22.8	7.6	44.3	16
PCB-138	0.21	0.34	2.36	1.2	15.4	8.7	24.0	28
PCB-153	0.19	0.34	2.32	1.5	14.0	8.7	23.5	36
PCB-180	0.03	0.06	0.33	0.5	2.2	1.5	3.3	12

^1^ PCB number 28, 52, 101, 118, 138, 153 and 180.

^2^ Based on hay with 12% moisture content.

^3^ Average of 2 samples.

^4^ Average of 10 samples.

**Table 5:** Comparison between different dioxin-like PCB congeners in milk and hay form Engidalur with background levels. Both absolute concentrations and the relative contributions of TEQ of each congener are presented.

	Hay (pg/g)^1^		Milk (pg/g fat)		Hay (% of PCB WHO-TEQ)		Milk (% of PCB WHO-TEQ)	
	Engidalur	Control C^2^	Engidalur	Control C^3^	Engidalur	Control C^2^	Engidalur	Control C^3^
	
Non-ortho PCBs								
PCB-77	28	17	6,8	<3	0.55	1.4	0.02	<0.1
PCB-81	1.8	1.1	3,0	<0,2	0.04	0.1	0.01	<0.01
PCB-126	4.4	0.5	35,5	2,7	86.1	40.8	81.0	77.8
PCB-169	0.77	<0.09	7,5	0,7	1.5	<0.7	1.7	2.0
Mono-ortho PCBs								
PCB-105	130	88	1071	146	2.5	7.2	2.4	4.2
PCB-114	7	8	118	17	0.69	3.3	1.3	2.4
PCB-118	310	452	4358	10	6.1	36.9	9.9	0.3
PCB-123	<5	11	80	<11	<0.02	0.9	0.2	<0.3
PCB-156	22	18	231	67	2.2	7.3	2.6	9.7
PCB-157	3	3	56	19	0.29	1.2	0.6	2.7
PCB-167	5	9	107	55	0.01	0.1	0.02	0.2
PVB-189	1	<2	17	<10	0.02	<0.2	0.04	<0.3

^1^ The results are based on hay with 12% moisture content.

^2^ Average of 4 samples.

^3^ Average of 10 samples.

## Discussion

The current study suggests that the operation of a small municipal waste incinerator, not satisfying modern day emission standards, may result in non-compliant levels of dioxins and dioxin-like PCBs in locally produced foods. The incident was limited to a small area were non-compliant levels in milk and beef were observed. Our results do, however, demonstrate the difficulty of evaluating contaminant levels in lambs and ewes, which could migrate freely in and out of the contaminated area.

When evaluating the contamination in Engidalur, the milk samples were considered most reliable. Firstly, contaminant levels in milk are known to be closely correlated with levels in adipose tissue [24] and since the samples were collected from the milk tank at the farm, they should reflect average contaminant levels in the herd. Secondly, the herd was located at the farm only and was predominantly fed on hay harvested in the valley. The hay sample, which was a composite sample from different bales of hay harvested during the summer, showed PCDD/Fs levels that were at least seven times higher than background values (0.85 versus 0.12 pg total WHO-TEQ/g). It is worth noting in this context that the PCDD/Fs levels in the control sample of hay were most likely lower than the upperbound level of 0.12 pg WHO-TEQ/g, as most congeners in the control sample were below limit of quantification. From these results it was concluded that the source of the contamination was due to deposition of contaminated fly ash from the nearby incinerator.

The congener pattern of PCDD/Fs was similar for the milk and hay samples from Engidalur with 2,3,4,7,8-PeCDF accounting for 30% and 36% of PCDD/Fs WHO-TEQ in hay and milk, respectively. The corresponding numbers for the control samples were <15% and 1.5% for hay and milk, respectively. Similarly, the 1,2,3,7,8 PeCDD accounted for 23% and 34% of PCDD/Fs in hay and milk from Engidalur but the average contribution for the control milk sample was <3.4%. This pattern of approximately 50% of the total PCDD/Fs contribution from 2,3,4,7,8-PeCDF and 1,2,3,7,8 PeCDD is not fully consistent with the pattern observed in the recent incident in Italy [17] were the source of the contamination was also considered to be burning of waste. In contrast to our setting, the Italian incident was most likely related to open burning of waste while in our case the source can be considered more controlled (although not up to date with modern standards). Studies have also shown that the congener patter depends of the type of material being burned as well as the temperature [25,26], which may explain the unique pattern that appears to occur in each food contamination incident [12-14,17].

Unlike the case for the PCDD/Fs, the marker PCBs (Table 4) show a marked difference in relative distribution between the hay and the milk, where the contribution of the lighter PCBs (PCBs #52, 52 and 101) is much higher in the hay than in milk indicating different uptake and/or elimination routes of light versus heavy PCBs in the milking cows. This is supported by the fact that the relative distribution of marker PCBs is similar in milk of both control sample and the samples from Engidalur in spite of the fact that the marker PCBs in the control hay-samples have a greater contribution of the lighter PCBs than in Engidalur. The greater contribution of lighter PCBs in the control hay indicates long-range atmospheric transport in the background samples while a more local source seems to be affecting the sample in Engidalur. It is noticeable that the levels of marker PCBs in the hay are somewhat higher in the control samples than in the samples from Engidalur, most likely reflecting both temporal and spatial trends.

The congener pattern of dioxin-like PCBs is similar for the milk and hay samples from Engidalur with contribution of PCB-126 dominating with 86 and 81% of the PCBs’ WHO-TEQ, respectively (Table 5). The relative distribution of dioxin-like PCBs in hay from Engidalur differs from the background in mostly higher contribution from PCB-126 in Engidalur, *i.e.* by 86% and 41% for Engidalur and control, respectively. The concentration differences in dioxin-like PCBs in Engidalur and control in terms of TEQ differ considerably or by a factor of about 5. Unlike the relative distribution of non- and mono-ortho PCBs in hay, the relative distribution of dioxin-like PCBs in milk from Engidalur and milk from the background is fairly similar where the main contribution to the WHO-TEQ in both is from PCB-126 or 80%. The concentration difference in milk for the dioxin-like PCBs in terms of TEQ is, however, large or about 11-fold.

Dioxins and PCBs have high affinity to organic matters in soils and sediments, compartments that are regarded as major sinks and in which slow degradation takes place. Transfer of dioxins from soil to plants is considered very limited but grazing animals and humans may be exposed to dioxins and PCBs through contaminated soil particles on plant surfaces or by wind erosion [27]. The soils sampled in relation to the current incident have previously been described as Histic Andosols [28] which generally contain 12-20% organic carbon. This fact suggests that PCDD/Fs and PCBs accumulating in theses soils may be strongly bound within the soil system. Moreover, the climate of the region has been characterized as sub-arctic [29], which results in slower natural degradation of the deposited PCDD/Fs and PCBs onto these soils due to both relatively low exposure to degrading solar light for long periods of time and due to relatively slow rate of volatilization into air where photolysis is faster. The upperbound levels of PCDD/Fs in soils collected beneath the grass turf in Engidalur were below 5 pg I-TEQ/g d.m. for all but one sample (5.3 pg I-TEQ/g d.m.) of the 9 samples collected [19] . When levels of dioxins in soil are below 5 pg I-TEQ/g d.m., restrictions are generally not imposed on agricultural activities [30]. However, the main limitation of the soil sampling programme, preventing unambiguous conclusions to be drawn, is the lack of information on the PCDD/Fs levels within the grass turf layer. The grazing area is usually not ploughed and given the high affinity of dioxins to organic matter, the grass turf layer might have contained elevated concentrations, particularly at the immediate surface.

With annual production of milk and meat in Engidalur of 45 and 6 tons, respectively, the dioxin incident reported in this paper cannot be considered as significant in terms of the amount of contaminated foods distributed or the number of consumers affected. Location of the incinerator in a narrow valley at bottom of a fjord with approximately 700m high mountains on each side results in prevailing calm weathers and thus appears to have limited the contamination to a relatively small area. The levels of PCDD/Fs and dioxin-like PCBs in milk from nearby fjords support that conclusion.

As soon as the contamination was discovered in Engidalur, collection of milk from the farm was stopped and delivery of animals to slaughter was prohibited. As a result of this incident, the incinerator was closed down in early 2011 by the relevant authority. Furthermore, all food products on the market that could possibly be contaminated, both in Iceland and in Europe, were withdrawn and disposed of. An expert panel was established by The Icelandic Food and Veterinary Authority to provide a scientific opinion on the utilization of the animal products in the area and the future possibilities for agricultural activities in the area. As a result of the opinion of the ad hoc working group, all animals used for food production from the two farms in question were culled and all potentially contaminated feed has been disposed of. This decision is in slight contrast to the recent incident in Italy were non-compliant herds from around 100 farms were monitored at 45 day intervals until compliant levels in milk were observed [17]. In the current case the number of potentially contaminated animals was much smaller and this incident was not confined to dairy cows only. Using similar approach as in Italy would have been considerably more expensive and potentially less effective than culling the animals.

Following up on this incident and in the light of divergent results on hay and soil samples, a grazing experiment is currently being conducted to evaluate whether the uptake of dioxins and dioxin-like PCBs still occurs from plants or from the surface layer of the soils in Engidalur. The results of that experiment will be used for determining next steps with respect to the agricultural activities in the area.

The incinerator in Engidalur was new when it first started its operation in 1995 and can therefore not be considered as a typical “old” incinerator often associated with elevated dioxins emissions [31]. The incinerator did, however, receive dispensation from the EU regulations on maximum limit of dioxins in fly ash of 0.1 ng I-TEQ /m^3^. The argument for seeking dispensation was on one hand the relatively high cost of meeting this requirement for a unit with such a small throughput (≈3000 tons waste/year) and on the other hand it was considered unlikely that such a small incinerator could have a significant impact on the local surroundings. That decision was not reassessed after the inspection of the incinerator in 2007 which showed elevated emission levels. Based on that single measurement, the annual emission of dioxins from the incinerator was estimated to be 0.087 g I-TEQ. The current incident does therefore support the EU regulation on dioxins emissions from incinerators and demonstrates that dispensation from the regulation may result in contamination of the environment. Dispensation should at least be followed up by a continuous monitoring and inspection of the nearby surroundings to ensure its rationale.

## Summary

Although limited in scope, the current incident clearly demonstrates that operation of a small waste incinerator that is non-compliant with the current EU legislation may result in elevated levels of dioxins in foods in the nearby surroundings. This incident also demonstrates the difficulty of tracing contaminant sources by migratory animals such as sheep that can freely move in and out of the contaminated area. With respect to future monitoring programs conducted by the Icelandic Food and Veterinary Authority, the incident highlights the need of good flow of information between different inspection authorities; and the importance of targeted monitoring of food production close to potential sources of contamination, even though the sources may be relatively small in scale.

## Competing interests

The authors declare that they have no competing interests.

## Authors' contributions

KH and SOH designed the experiment and collected the samples. TIH, GAA, OD, and RG provided expert advice during the experiment and with respect to interpretation of the results. TIH and GAA drafted the manuscript with input and critical revisions from KH, SOH, OD and RG.
